# Medium-thickness-dependent proton dosimetry for radiobiological experiments

**DOI:** 10.1038/s41598-019-48100-9

**Published:** 2019-08-09

**Authors:** Mehrdad Shahmohammadi Beni, Dragana Krstic, Dragoslav Nikezic, Kwan Ngok Yu

**Affiliations:** 1 Department of Physics, City University of Hong Kong, Tat Chee Avenue, Kowloon Tong, Hong Kong; 2 Faculty of Science, University of Kragujevac, Kragujevac, Serbia; 3 State Key Laboratory in Marine Pollution, City University of Hong Kong, Tat Chee Avenue, Kowloon Tong, Hong Kong

**Keywords:** Biophysics, Software

## Abstract

A calibration method was proposed in the present work to determine the medium-thickness-dependent proton doses absorbed in cellular components (i.e., cellular cytoplasm and nucleus) in radiobiological experiments. Consideration of the dependency on medium thickness was crucial as the linear energy transfer (LET) of protons could rise to a sharp peak (known as the Bragg peak) towards the end of their ranges. Relationships between the calibration coefficient *R* vs medium-layer thickness were obtained for incident proton energies of 10, 15, 20, 25, 30 and 35 MeV, and for various medium thicknesses up to 5000 μm, where *R* was defined as the ratio *D*_*A*_*/D*_*E*_, *D*_*A*_ was the absorbed proton dose in cellular components, and *D*_*E*_ was the absorbed proton dose in a separate radiation detector. In the present work, *D*_*A*_ and *D*_*E*_ were determined using the MCNPX (Monte Carlo N-Particle eXtended) code version 2.4.0. For lower incident proton energies (i.e., 10, 15 and 20 MeV), formation of Bragg-peak-like features were noticed in their *R*-vs-medium-layer-thickness relationships, and large *R* values of >7 and >6 were obtained for cytoplasm and nucleus of cells, respectively, which highlighted the importance of careful consideration of the medium thickness in radiobiological experiments.

## Introduction

Many experimental data were accumulated in literature with the ultimate goal of revealing the biological effects of protons with various energies for different types of cells. Monoenergetic proton beams employed in radiobiological experiments provided useful information on the energy dependence of biological effectiveness, especially at low energies where the relative biological effectiveness (RBE) was larger than unity^1^. Monoenergetic proton beams with energy lower than the energies commonly employed in clinical proton therapy were previously used to systematically study the inactivation of mammalian cells (mostly rodent cells)^2–8^. Belli *et al*.^1^ also studied the inactivation of human cells (both normal and tumor cells) exposed to protons with energies lower than those used in clinical proton therapy. The biological effects of protons with energies from 5 to 35 MeV were studied on *in-vitro* irradiated cell lines of human origin^7^, beagle eyes^9^ and on whole body of the primates^10^.

For such radiobiological experiments, accurate dosimetry for these protons in the cells or the cellular components (such as the cell nuclei) would be crucial for establishing realistic dose-response relationships and for meaningful comparisons among different types of radiations (e.g., proton and photon beams). Using an external radiation detector (i.e., PTW TM31013 ionization chamber with PTW UNIDOS electrometer, calibrated in the secondary standard laboratory at the Central Office of Measures in Warsaw, Poland), 1 to 5 Gy of proton dose was delivered to uveal melanoma (Mel270) and skin melanoma (BLM) cell lines and their respective cell survival curves were constructed^11^. Details regarding beam dosimetry were taken from the TRS-398 protocol recommended by International Atomic Energy Agency, with a reference dosimeter of a PTW TM31010 semiflex ionization chamber and a PTW UNIDOS Webline electrometer (PTW, Freiburg, Germany). Nohtomi *et al*.^12^ studied the response of a small ionization chamber (sensitive volume of 0.01 ml) with proton beams and compared the results with JARP (Japanese Association of Radiological Physicists) ionization chambers (sensitive volume of 0.6 ml) that were commonly called thimble-type chambers. In a separate study, the absorbed dose of protons was determined using seven different types of ionization chambers, namely, two plane parallel (NACP-02 and Roos FK-6) and five cylindrical (three NE-2571 and two M IC-18) ones^13^. Dhanesar *et al*.^14^ pointed out that measurements of the percentage depth dose (PDD) for proton beams were mostly accomplished by a water tank dosimetry system with ionization chamber.

Previously, Auer *et al*.^15^ irradiated a HeLa cell monolayer using a 20 MeV proton beam and delivered a dose of 3 Gy which was theoretically calculated from the current density of the beam^16^. In addition, 5 and 10 Gy of proton and photon doses were delivered to glioma stem cells (GSCs) and the cellular responses to both proton and photon beams were investigated^17^. In that study^17^, the cells were irradiated in the region of interest within a custom-made solid water phantom and the proton dose was calculated using the depth at which the region of interest was located. It was found that a proton beam was more effective to accomplish successful cell killing when compared to a photon beam. Although proton-induced cell death mechanisms were extensively studied and reported^18–21^, proton-induced biological effects were still not fully understood^18^. In some researches, their biological effects were assumed to be similar to those induced by photons^22^. However, some experimental data showed that such an assumption was not always valid^23,24^.

An issue in accurate dosimetry for protons in radiobiological experiments was the dependence of medium thickness on the proton dose delivered to cells or cellular components (such as the cell nuclei). As a proton loses its energy along its track while propagating through matter, its linear energy transfer (LET) increases. As such, the proton dose delivered to cells or cellular components was expected to be critically dependent on the medium thickness. Surprisingly, this issue was not examined extensively in the literature, and only very few research groups actually reported the medium thickness in their experiments. An example was found in the literature reporting that the cells were irradiated underneath 12.5 cm of solid water using a proton beam (where the Bragg peak was formed)^17^. The main objective of the present work was to study medium-thickness-dependent proton doses (for proton energies *E*_*proton*_ = 5 to 35 MeV) in radiobiological experiments, and to provide a feasible calibration for proton irradiations in radiobiological experiments with different medium thickness.

Direct measurements of absorbed doses in cells would be formidable, although direct measurements had been performed using traditional solid state-nuclear track detectors and fluorescent nuclear track detectors^25–27^. Instead of direct measurements, these doses were commonly obtained through computer simulations. For example, Jia *et al*.^28^ developed a Monte Carlo model for studying irradiation of a slab head phantom with proton beams with varying energies, which enabled accurate determination of proton doses for modeled brain tissues in the slab head phantom. For radiobiological experiments, the doses absorbed in the studied cells were commonly surrogated by the doses recorded using an external radiation detector (e.g., ionization chamber), which would however require an accurate conversion coefficient *R* (=*D*_*A*_*/D*_*E*_), where *D*_*A*_ was the dose absorbed in exposed cells or cellular components while *D*_*E*_ was the dose recorded by the external detector. Determination of this conversion coefficient *R* was in fact the focus of our recent studies on the calibration for realistic neutron^29^ and photon^30^ dosimetry in radiobiological experiments. Based on these previous works, the present task was to enable quantification of the absorbed dose (*D*_*A*_) in exposed cells or cellular components due to protons through the dose (*D*_*E*_) through the conversion coefficient *R*, the value of which depended on the medium thickness.

## Material and Methods

**Figure 1:**
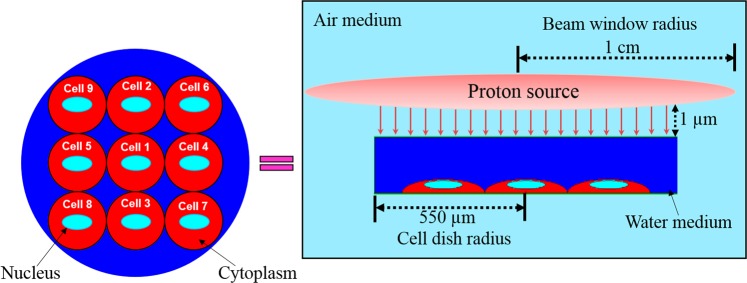
Schematic representation of the modeled cell array containing nine cells covered with a cylindrical medium (composed of water) and exposed to a proton beam.

Mukherjee *et al*.^31^ and references therein studied the biological effects induced in cells by protons with energies from 5 to 35 MeV. Similarly, we considered incident protons with discrete energies of 10, 15, 20, 25, 30 and 35 MeV in the present work. The present model was built using the MCNPX (Monte Carlo N-Particle eXtended) code^32^. The transport and interaction events of three different particles were considered, namely, primary protons, secondary neutrons and photons^28^. An array of cells consisting of nine identical cells with separate cytoplasm and nucleus domains were modeled in the present work, the arrangement of which is shown in Fig. 1. The arrangement of the cells in the array followed the work of Clarke and Jevremovic^33^, where the nine cells were arranged in a lattice structure and covered by a medium (water) layer. The modeled cells were half ellipsoids with semi-major, semi-mean and semi-minor axes of 5, 15 and 15 µm, respectively, while the cell nuclei were modelled as ellipsoids with semi-major, semi-mean and semi-minor axes of 1.5, 7 and 7 µm, respectively *cf*.^33^. The composition of cell cytoplasm was taken as 59.6% ^1^H, 24.24% ^16^O, 11.11% ^12^C, 4.04% ^14^N and 1.01% ^31^P, while that of the cell nucleus was adopted as 10.64% ^1^H, 74.5% ^16^O, 9.04% ^12^C, 3.21% ^14^N and 2.61% ^31^P, and the medium was assumed to be pure water. The irradiation dish was modeled as a pure water cylinder with a radius of 550 µm and with variant heights (the height corresponding to the thickness of water medium layer). The cells were placed inside the cylindrical water dish on a thin cylindrical glass (SiO_2_) substrate with a thickness of 0.2 µm. Similar to our previous works^29,30^, a uniform disk source (with a radius set as 1 cm) was used to irradiate the cells that were located underneath of the medium layer. The thickness of the medium layer was chosen to be 100, 500, 1000, 1500, 2000, 2500, 3000, 3500, 4000, 4500 and 5000 μm.

In the present work, a very small source-to-target distance of 1 µm (i.e., air thickness) was chosen to avoid proton attenuation and energy loss in the surrounding air. It was noted that Jia *et al*.^28^ also placed the proton source close to the modeled slab head phantom for a better conformation of the dose distribution in the target slab. The irradiation setup employed here to determine the absorbed dose (*D*_*A*_) in exposed cells (in both cytoplasm and nucleus) is shown schematically in Fig. 1. The results were obtained for the central cell (number 1), side cells (number 2 to 5) and diagonal cells (number 6 to 9) cells to check for potential differences. The results for the side and diagonal cell groups were represented by arithmetic means for the corresponding groups.

**Figure 2:**
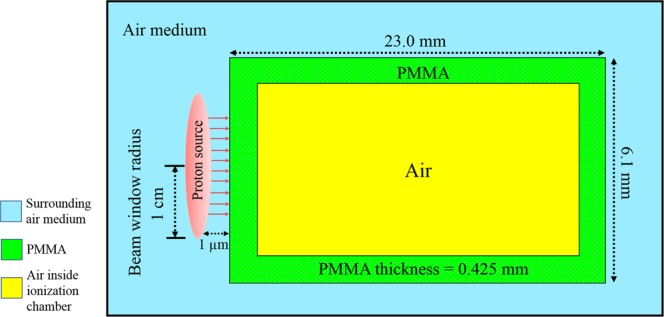
Schematic representation of the proton irradiation of the ionization chamber.

The proton dose (*D*_*E*_) recorded by a detector located external to the targeted cells determined by the thimble ionization chamber (IOC) (model TN 30013, waterproof PTW Farmer^®^ Chamber) which was connected to the PTW UNIDOS^®^ E Universal Dosemeter (SN006861, PTW, Freiburg, Germany). The IOC had a cylindrical geometry (radius = 3.05 mm; length = 23.0 mm) with a sensitive volume of ~0.5 cm^3^. The wall layer of the IOC was made of 0.425 mm thick PMMA (also known as acrylic, with a density of 1.18 gcm^–3^) with atomic composition comprising 33.3% of ^12^C, 13.3% of ^16^O and 53.4% of ^1^H. The proton dose in the IOC was determined for different incident proton energies, namely, 10, 15, 20, 25, 30 and 35 MeV. The schematic diagram shown in Fig. 2 describes the irradiation setup for determination of *D*_*E*_.

The dose was determined using the track length estimate of energy with the unit of MeV/g (tally F6 of the MCNPX code) for each specific particle. The dose *D*_*A*_ absorbed in the cell cytoplasm or nucleus, and the dose *D*_*E*_ recorded by the external detector were determined through the track length estimate of energy in the corresponding cellular component and the IOC, respectively, using the same tally. The conversion coefficient *R* was then computed as *R* = *D*_*A*_/*D*_*E*_. The determined dose values were normalized by the emitted source particles and therefore explicit evaluation of particle flux (*Φ*) was not necessary to construct the calibration coefficients *R*. We refer interested readers to our previous works^29,30,34^ and references therein for more details about the development and the practical use of *R*. The Vavilov model was used to explain the charge particle (for protons) straggling, and the neutron and photon cross-section data were taken from ENDF/B-VII and ENDF/B-VI release 8 photoatomic data, respectively. In addition, the code was executed using the MCNPX version 2.4.0.

## Results and Discussion

### Absorbed proton dose in IOC (*D*_*E*_)

**Figure 3:**
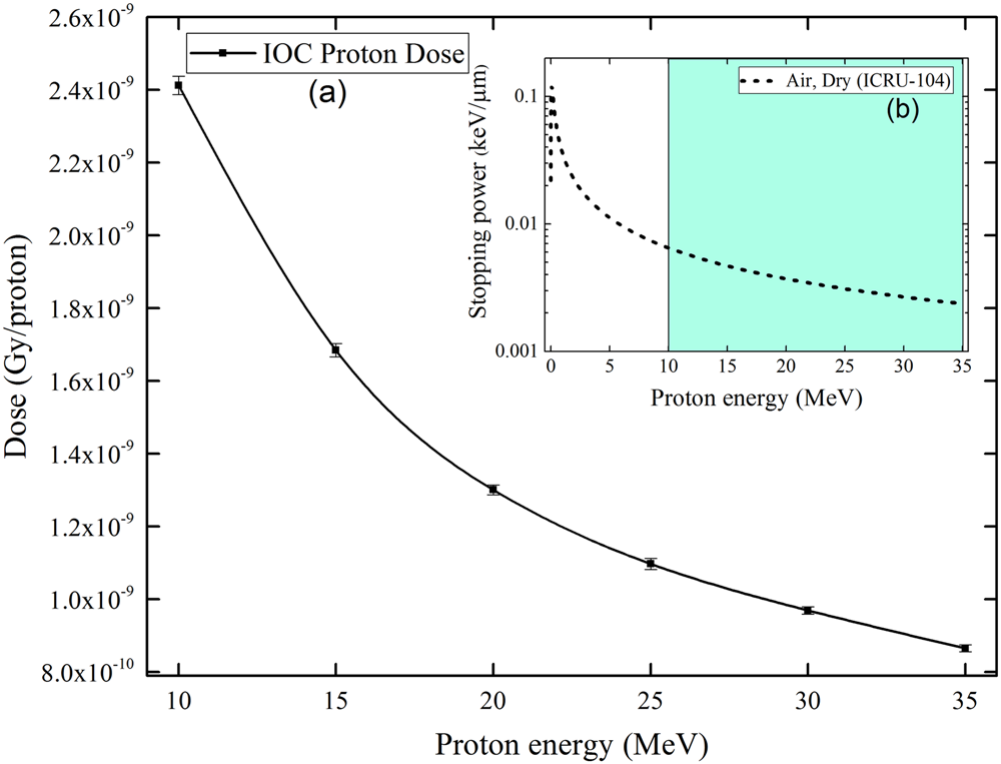
(**a**) Proton dose deposited in the sensitive volume of ionization chamber from protons with different incident energies. (**b**) Stopping power of protons in dry air as a function of incident proton energy (the green region indicates the energy range of incident protons considered in the present work).

Figure 3 shows the proton dose deposited in the IOC sensitive volume (*D*_*E*_) from different incident proton energies. For smaller incident proton energies, *D*_*E*_ increased because of the larger stopping power (see Fig. 3b) that led to larger fractions of deposited energies in the IOC sensitive volume.

### Absorbed proton dose in cells and *R* values

**Figure 4:**
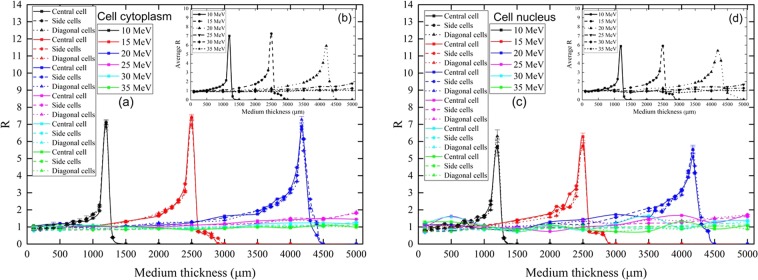
Variations of (**a**) calibration coefficient *R* and (**b**) average *R* with medium thickness in the cell cytoplasm, and variations of (**c**) *R* and (**d**) average *R* with medium thickness in the cell nucleus, for different incident proton energies. (The lines connecting the points do not represent data and are only presented to help the eye). (Note: error bars are smaller than the symbols).

The absorbed proton doses in cellular components (i.e., cellular cytoplasm and nucleus) (*D*_*A*_) were determined for different medium layer thicknesses. The calibration coefficients *R* were determined for three different groups of cells, namely, the central, side and diagonal cells. The variation of *R* versus different medium thicknesses for different incident proton energies are shown in Fig. 4.

For larger proton energy (when the proton energy was larger than the energy corresponding to the Bragg peak), the proton dose deposited in matter became smaller^28^, which was due to the lower stopping power of the protons. In the present irradiation setup, the cells were placed below a medium layer with varying thicknesses, so the protons launched from the source needed to propagate through the medium layer before reaching the cells and to interact with the cellular components (i.e., cytoplasm and nucleus). While propagating through the medium, the protons lost their energies due to interactions, and their stopping power increased. As such, the proton doses deposited in the cell cytoplasm and nucleus increased with the medium layer thickness. Similar trends were observed for *R* with the medium thickness for both cytoplasm (Fig. 4a) and nucleus (Fig. 4c).

For lower incident proton energies (i.e., 10, 15 and 20 MeV), formation of Bragg-peak-like features were noticed in their *R*-vs-medium-layer-thickness relationships. For example, for 15 MeV protons, the dose delivered to cellular components (i.e., cytoplasm and nucleus) would start to increase up to ~2500 µm of medium layer thickness, which was attributed to the higher doses delivered to the cytoplasm and nucleus for lower energy protons (that were slowed down in the medium layer). However, employment of thicker medium layers could also prevent the protons from reaching the cells. For 15 MeV protons, the incident protons could not reach the cellular components when the medium layer was thicker than ~2500 µm. Therefore, the *R*-vs-medium-layer-thickness relationship displayed a Bragg-peak-like feature. It was interesting to note that variations in the absorbed proton doses in the cellular components (i.e., cytoplasm and nucleus) was negligible for protons with the same incident energy, which demonstrated the robustness of the methodology proposed in this work. The *R* values could be applied to calibrate the dose when constructing the survival curve, which has a particular importance in radiobiological experiments. In fact, in one of our previous works^30^, the *R* value was used to calibrate the dose for the normalized mean apoptotic events in zebrafish embryos induced by 150 kV X-rays with different levels of hardness. The concept and methodology can also easily be extended to studies with different detectors, different targets and different energies of ionizing radiations.

The *R* values for medium thicknesses lower than those corresponding to the peak *R* values were close to unity, which happened for both cell cytoplasm and nucleus. For more convenient observation of the relationship among *R*, proton energy and medium thickness, the “average” *R* values were shown in Fig. 4(b,d), which were computed as the arithmetic mean of all *R* values for the nine cells in the array for the specified proton energy and medium thickness. Considering the average *R* values, for incident proton energies of 10, 15 and 20 MeV, the largest *R* value was obtained at 1000, 2500 and 4000 µm medium layers, respectively. Further increase in the thickness of the medium layer shielded off the protons from reaching the underneath cells, and no more proton energy could be deposited in the cytoplasm and nucleus. In contrast, considering the proton energies of 25, 30 and 35 MeV, the *R* values were rather close to unity in both cytoplasm and nucleus. The largest *R* values in the cytoplasm were found as 1.78, 1.26 and 1.04 for incident proton energies of 25, 30 and 35 MeV, respectively. Conversely, the largest *R* values in the nucleus were found as 1.66, 1.24 and 0.98 for incident proton energies of 25, 30 and 35 MeV, respectively. In this energy range, the protons had relatively larger ranges in the medium layer (>5000 µm). Therefore, even the largest thickness of the medium layer (5000 µm here) was not capable to significantly reduce these proton energies to exhibit the formation of Bragg-peak-like features in the *R*-vs-medium-layer-thickness relationships.

**Figure 5:**
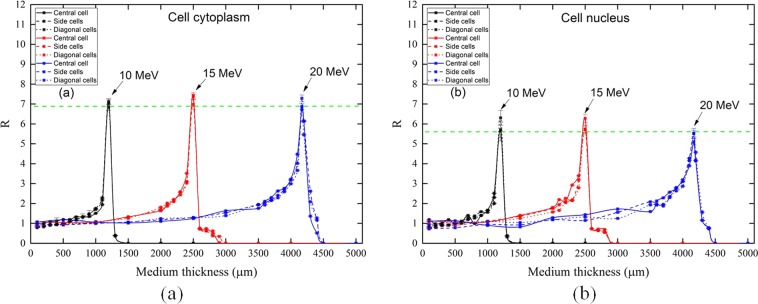
Variations of calibration coefficient *R* in (**a**) cytoplasm and (**b**) nucleus of cells for proton with incident energies of 10, 15 and 20 MeV, showing the maximum *R* values. (The lines connecting the points do not represent data and are only presented to help the eye). (Note: error bars are smaller than the symbols).

The range of 15 MeV protons in water was close to 2500 μm, so protons exiting from a water medium thickness of 2500 μm (which could still reach the cellular components) would practically have the lowest energies and thus highest stopping power, and as a result transferred the maximum energy to the cytoplasm and nucleus (see Fig. 4). Simply put, when the range of protons was larger than (water medium layer thickness) and smaller than (water medium layer thickness + cell thickness), we could observe such a large *R* value. For a comparison, the range of 10 MeV protons in water was close to 1200 μm (which fell between 1000 and 1500 μm), so at 1000 μm maximum energy transfer to cellular components could not take place since the protons would pass through the cellular components. In contrast, the range of 20 MeV protons in water was close to 4170 μm (which fell between 4000 and 4500 μm). For 4000 μm, again full energy transfer could not occur, and for 4500 μm all protons would be stopped in the water medium layer. To further elucidate the phenomenon, the variations of *R* in cellular components for protons with incident energies of 10, 15 and 20 MeV was compared in Fig. 5(a,b).

In summary, if the water medium thickness criterion for maximum energy transfer (range of protons was larger than water medium layer thickness and smaller than water medium layer thickness + cell thickness) was satisfied (i.e., ~1200, ~2500 and ~4170 µm for 10, 15 and 20 MeV, respectively), which corresponded to the Bragg peak in the stopping-power curve, large *R* values would be obtained as shown in Fig. 5(a,b), reaching values >7 and >6 for cytoplasm and nucleus of cells, respectively.

The results presented above in Figs 4 and 5 highlighted the importance of careful consideration of the medium thickness in radiobiological experiments, and provided guidance for choosing medium thicknesses which could lead to satisfactory surrogate of the doses *D*_*A*_ absorbed in cellular components by the doses *D*_*E*_ reported by the external detector, i.e., *R* = *D*_*A*_*/D*_*E*_ ≈ 1. From Figs 4 and 5, it was noticed that to satisfy this condition could be a challenge particularly for protons with lower energies due to occurrence of Bragg peaks at relatively small medium layer thicknesses, e.g., ~1200 µm for 10 MeV protons. If such low-energy protons had to be used for radiobiological experiments, the medium layer thickness should be carefully monitored and, if necessary, appropriate corrections to the doses *D*_*A*_ absorbed in cellular components should be performed accordingly, i.e., *D*_*A*_ should be computed by *R× D*_*E*_ (with *R* values given in Figs 4 and 5) instead of simply taking *D*_*A*_ = *D*_*E*_.

## Conclusions

A calibration method was proposed in the present work for proton dosimetry for radiobiological experiments, which was medium-thickness-dependent. Relationships between *R* vs medium-layer thickness were obtained for incident proton energies of 10, 15, 20, 25, 30 and 35 MeV, and for medium thicknesses of 100, 500, 1000, 1500, 2000, 2500, 3000, 3500, 4000, 4500 and 5000 μm. For lower incident proton energies (i.e., 10, 15 and 20 MeV), formation of Bragg-peak-like features were noticed in their *R*-vs-medium-layer-thickness relationships, and large *R* values of >7 and >6 were obtained for cytoplasm and nucleus of cells, respectively, which highlighted the importance of careful consideration of the medium thickness in radiobiological experiments. The present calibration method would be useful for future radiobiological studies which would need precise dose values. Another direction to further improve the accuracy of the calibration method proposed in this work would be the development of more realistic models for cells employed in realistic radiobiological experiments.

## Data Availability

The datasets generated and analyzed during the current study are available from the corresponding author on reasonable request.

## References

[1] Belli D (2000). Inactivation of human normal and tumour cells irradiated with low energy protons. Int. J. Radiat. Biol..

[2] Perris A, Pialoglou P, Katsanos AA, Sideris EG (1986). Biological effectiveness of low energy protons. I. Survival of Chinese hamster cells. Int. J. Radiat. Biol. Relat. Stud. Phys. Chem. Med..

[3] Belli M (1989). RBE-LET relationship for the survival of V79 cells irradiated with low energy protons. Int. J. Radiat. Biol..

[4] Belli M (1993). Inactivation and mutation induction in V79 cells by low energy protons: re-evaluation of the results at the LNL facility. Int. J. Radiat. Biol..

[5] Folkard M (1989). The irradiation of V79 mammalian cells by protons with energies below 2 MeV: part I: experimental arrangement and measurements of cell survival. Int. J. Radiat. Biol..

[6] Folkard M (1996). Inactivation of V79 cells by low-energy protons, deuterons and helium-3 ions. Int. J. Radiat. Biol..

[7] Bettega D (1979). Relative biological effectiveness for protons of energies up to 31 MeV. Radiat. Res..

[8] Bettega D (1998). Inactivation of C3H10T1/2 cells by low energy protons and deuterons. Int. J. Radiat. Biol..

[9] Horn PL, Shifrine M (1973). Proton Irradiation of Beagle Eyes: II. Clinical and Histopathologic Observations for 35 MeV Protons. Radiat. Res..

[10] Boles LA, Blake KR, Parker CV, Nelson JB (1969). Physical dosimetry and instrumentation for low-energy proton irradiation of primates. Radiat. Res..

[11] Jasińska-Konior K (2017). Proton beam irradiation inhibits the migration of melanoma cells. PloS ONE.

[12] Nohtomi A, Sakae T, Tsunashima Y, Kohno R (2001). Dosimetry of pulsed clinical proton beams by a small ionization chamber. Med. Phys..

[13] Medin J (1995). Ionization chamber dosimetry of proton beams using cylindrical and plane parallel chambers. Nw versus NK ion chamber calibrations. Phys. Med. Biol..

[14] Dhanesar S (2013). Quality assurance of proton beams using a multilayer ionization chamber system. Med. Phys..

[15] Auer S (2011). Survival of tumor cells after proton irradiation with ultra-high dose rates. Radiat. Oncol..

[16] Dollinger G (2009). Nanosecond pulsed proton microbeam. Nucl. Instrum. Methods. Phys. Res. B.

[17] Mitteer RA (2015). Proton beam radiation induces DNA damage and cell apoptosis in glioma stem cells through reactive oxygen species. Sci. Rep..

[18] Di Pietro C (2006). Cellular and molecular effects of protons: apoptosis induction and potential implications for cancer therapy. Apoptosis.

[19] Ianzini R, Cherubini MA, Mackey F (1999). Mitotic catastrophe induced by exposure of V79 Chinese hamster cells to low-energy protons. Int. J. Radiat. Biol..

[20] Lee KB, Lee JS, Park JW, Huh TL, Lee YM (2008). Low energy proton beam induces tumor cell apoptosis through reactive oxygen species and activation of caspases. Exp. Mol. Med..

[21] Lee KB, Kim KR, Huh TL, Lee YM (2008). Proton induces apoptosis of hypoxic tumor cells by the p53-dependent and p38/JNK MAPK signaling pathways. Int. J. Oncol..

[22] Kedracka-Krok S (2014). Proteomic analysis of proton beam irradiated human melanoma cells. PloS ONE.

[23] Girdhani S, Sachs R, Hlatky L (2013). Biological effects of proton radiation: what we know and don’t know. Radiat. Res..

[24] Gridley DS (2011). Comparison of proton and electron radiation effects on biological responses in liver, spleen and blood. Int. J. Radiat. Biol..

[25] Kodaira S (2019). Evidence of local concentration of α-particles from 211At-labeled antibodies in liver metastasis tissue. J. Nucl. Med..

[26] Konishi T, Kodaira S, Itakura Y, Ohsawa D, Homma-Takeda S (2018). Imaging uranium distribution on rat kidney sections through detection of alpha tracks using CR-39 plastic nuclear track detector. Radiat. Prot. Dosim..

[27] Kodaira S (2014). Co-visualization of DNA damage and ion traversals in live mammalian cells using a fluorescent nuclear track detector. J. Radiat. Res..

[28] Jia SB, Hadizadeh MH, Mowlavi AA, Loushab ME (2014). Evaluation of energy deposition and secondary particle production in proton therapy of brain using a slab head phantom. Rep. Pract. Oncol. Radiother..

[29] Shahmohammadi Beni M, Krstic D, Nikezic D, Yu KN (2016). A calibration method for realistic neutron dosimetry in radiobiological experiments assisted by MCNP simulation. J. Radiat. Res..

[30] Shahmohammadi Beni M, Krstic D, Nikezic D, Yu KN (2017). Realistic dosimetry for studies on biological responses to X-rays and γ-rays. J. Radiat. Res..

[31] Mukherjee B (1982). A simple irradiation facility for radiobiological experiments with low energy protons from a cyclotron. Nucl. Instr. Meth. Phys. Res..

[32] X-5 Monte Carlo Team. MCNP–a General Monte Carlo N-Particle Transport Code, Version 5. Vol. I: Overview and Theory. Los Alamos: Los Alamos National Laboratory. LA-UR-03-1987 (2003).

[33] Clarke SD, Jevremovic T (2005). MCNP5 evaluation of dose dissipation in tissue-like media exposed to low-energy monoenergetic X-ray microbeam. Radiat. Environ. Biophys..

[34] Shahmohammadi Beni M, Krstic D, Nikezic D, Yu KN (2018). Modeling kV X-ray-Induced Coloration in Radiochromic Films. Appl. Sci..

